# Maternal antenatal vitamin D supplementation and offspring risk of atopic eczema in the first 4 years of life: evidence from a randomized controlled trial

**DOI:** 10.1111/bjd.21721

**Published:** 2022-08-03

**Authors:** Sarah El‐Heis, Stefania D’Angelo, Elizabeth M. Curtis, Eugene Healy, Rebecca J. Moon, Sarah R. Crozier, Hazel Inskip, Cyrus Cooper, Nicholas C. Harvey, Keith M. Godfrey, the MAVIDOS Trial Group

**Affiliations:** ^1^ Medical Research Council Lifecourse Epidemiology Centre University of Southampton Southampton UK; ^2^ Dermatopharmacology, Faculty of Medicine University of Southampton Southampton UK; ^3^ NIHR Applied Research Collaboration Wessex Southampton Science Park, Innovation Centre Southampton UK; ^4^ NIHR Nutrition Biomedical Research Centre University of Southampton and University Hospital Southampton NHS Foundation Trust Southampton UK; ^5^ NIHR Musculoskeletal Biomedical Research Unit University of Oxford Oxford UK; ^6^ Developmental Sciences University of Southampton Southampton UK

## Abstract

**Background:**

Evidence linking prenatal maternal vitamin D supplementation with the offspring’s risk of atopic eczema is inconsistent, with most data coming from observational studies.

**Objectives:**

To examine the influence of maternal cholecalciferol supplementation during pregnancy on the risk of atopic eczema in the offspring at ages 12, 24 and 48 months.

**Methods:**

Within the UK Maternal Vitamin D Osteoporosis Study (MAVIDOS) double‐blind, randomized placebo‐controlled trial, we examined the relationship of maternal vitamin D supplementation during pregnancy with offspring atopic eczema at ages 12, 24 and 48 months. In MAVIDOS, pregnant women were allocated to either cholecalciferol 1000 IU per day or matched placebo, taken from around 14 weeks’ gestation until delivery, with the primary outcome of neonatal whole‐body bone mineral content. The prevalence of atopic eczema in the offspring was ascertained at ages 12 (*n* = 635), 24 (*n* = 610) and 48 (*n* = 449) months, based on the UK Working Party criteria for the definition of atopic dermatitis. The trial was registered with ISRCTN (82927713) and EudraCT (2007‐001716‐23).

**Results:**

The characteristics of mothers and offspring were similar between the intervention and placebo groups, apart from longer breastfeeding duration in the intervention group. Adjusting for breastfeeding duration, offspring of mothers who received cholecalciferol 1000 IU daily had a lower odds ratio (OR) of atopic eczema at age 12 months [OR 0·55, 95% confidence interval (CI) 0·32–0·97, *P* = 0·04]; this effect weakened and was not statistically significant at ages 24 months (OR 0·76, 95% CI 0·47–1·23) or 48 months (OR 0·75, 95% CI 0·37–1·52). The statistical interaction of intervention and breastfeeding duration in relation to eczema at age 12 months was not significant (*P* = 0·41), but stratification showed reduced infantile eczema risk in the intervention group for infants breastfed for ≥ 1 month (OR 0·48, 95% CI 0·24–0·94, *P* = 0·03) but not in those breastfed for < 1 month (OR 0·80, 95% CI 0·29–2·17, *P* = 0·66).

**Conclusions:**

Our data provide the first randomized controlled trial evidence of a protective effect of antenatal cholecalciferol supplementation on the risk of infantile atopic eczema, with the effect potentially being via increased breast milk cholecalciferol levels. The findings support a developmental influence on atopic eczema, and point to a potentially modifiable perinatal influence on atopic eczema.

**What is already known about this topic?**

- There are currently no antenatal interventions proven to reduce the incidence of infantile atopic eczema in the general population.
- However, observational studies have led to speculation that antenatal vitamin D supplementation may be beneficial.

## Graphical abstract

Our data, from the MAVIDOS double‐blind, randomized controlled trial, demonstrated a reduction in the risk of atopic eczema in infancy following maternal cholecalciferol supplementation during pregnancy; findings were weaker at ages 24 and 48 months. The protective effect of maternal cholecalciferol supplementation on infantile eczema was only significant in offspring breastfed for more than one month, but interaction terms between supplementation during pregnancy and breastfeeding duration were not statistically significant. The findings support a developmental influence on infantile atopic eczema and point to gestational cholecalciferol supplementation as a preventive strategy to reduce the burden of atopic eczema during infancy.

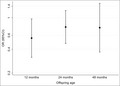

**Linked Comment:** D. Orchard. Br J Dermatol 2022; 187:630–631.

Atopic eczema is a chronic inflammatory condition that can substantially impact affected individuals, their families and the healthcare system. Estimated prevalences of atopic eczema have included 9·5% in children under age 4 years^1^ and 16·5% in children aged 1–5 years,^2^ with a rise observed globally over recent decades.^3^ There is increasing evidence that atopic eczema partly originates *in utero*, where genetic susceptibility and environmental exposures can affect the developing immune system and alter the skin barrier. Understanding the role of early‐life environmental exposures, such as maternal micronutrient status, may identify potential preventive strategies.

Inadequate gestational vitamin D status is highly prevalent in many populations.^4^, ^5^ Supplementation is recommended to prevent deficiency.^6^ Maternal serum levels of 25‐hydroxyvitamin D [25(OH)D] correlate with offspring levels at birth,^7^ and maternal vitamin D status has been extensively linked to offspring risk of atopic eczema and other atopic diseases, but with inconsistent evidence. One intervention study with high‐dose maternal antenatal vitamin D supplementation (2400 or 4000 IU daily) compared with placebo demonstrated a 25% reduction in the offspring’s risk of ‘asthma’ at age 0–3 years.^8^ Conversely, in an observational study, children born to mothers with late‐pregnancy serum 25(OH)D > 75 nmol L^−1^ had a higher risk of infantile eczema at age 9 months and childhood asthma age 9 years compared with children whose mothers had a concentration of < 30 nmol L^−1^.^9^ A trial in women at high risk of having offspring with asthma reported no significant difference in rates of offspring eczema at age 3 years following maternal antenatal supplementation with high (4400 IU daily) vs. low (400 IU daily) doses of vitamin D.^10^ Maternal vitamin D supplementation during pregnancy (2000 IU daily from 27 weeks’ gestation) increased vitamin D activity in breast milk,^11^ raising the possibility that benefits of gestational supplementation may arise from higher infant intakes after birth in supplemented mothers who breastfeed their infants.^12^

In this study, our aim was to examine the hypothesis that maternal supplementation with cholecalciferol 1000 IU daily during pregnancy would decrease the risk of atopic eczema in the offspring in the setting of a randomized controlled trial. We also sought to determine whether any associations varied by breastfeeding status and whether genetic variants previously associated with serum 25(OH)D concentrations^13^ were related to offspring atopic eczema.

## Patients and methods

Within the Maternal Vitamin D Osteoporosis Study (MAVIDOS), a multicentre, double‐blind, randomized placebo‐controlled trial, women were randomized to receive cholecalciferol 1000 IU daily or matched placebo, from 14 weeks’ gestation until delivery. The trial methods and primary findings have been published.^14^, ^15^ Pregnant women were invited to participate at their early‐pregnancy ultrasound screening appointment. Eligible women were recruited and randomized at 14 weeks’ gestation (or as soon as possible before 17 weeks’ gestation if recruited later) to either cholecalciferol 1000 IU daily or matched placebo [Merck KGaA, Darmstadt, Germany and Sharp Clinical Services (previously DHP‐Bilcare), Crickhowell, UK], taken until delivery. The sample size was determined for the primary outcome of neonatal whole‐body bone mineral content.

Inclusion criteria were women aged over 18 years, having a singleton pregnancy with a gestational age < 17 weeks based on last menstrual period and ultrasound measurements, and serum 25(OH)D between 25 and 100 nmol L^−1^ and calcium < 2·75 mmol L^−1^. Due to an ethics committee stipulation, only women with a baseline 25(OH)D of 25–100 nmol L^−1^ were eligible to participate. Women were excluded if they had known metabolic bone disease, renal stones, hyperparathyroidism or hypercalciuria; if they were taking medication known to interfere with fetal growth or more than 400 IU daily vitamin D supplementation; or if their fetus had a major anomaly. All participants received standard antenatal care, and could continue self‐administration of dietary supplements containing up to 400 IU daily vitamin D.

The MAVIDOS trial was conducted at three UK study sites – Southampton, Oxford and Sheffield – with a total of 965 births.^14^ Follow‐up for this study to examine the a priori hypothesis that maternal vitamin D supplementation would lower the incidence of atopic eczema was restricted to 703 Southampton children (352 intervention group and 351 placebo, Figure S1; see Supporting Information), who were assessed for eczema at ages 12 (*n* = 635), 24 (*n* = 610) and 48 (*n* = 449) months. Case definition was based on the UK Working Party diagnostic criteria for the definition of atopic eczema,^16^ assessed by trained research nurses who were blinded to intervention and control allocation and who performed a standardized questionnaire and examination. Itchy skin was a mandatory criterion in addition to three of: onset age < 2 years, history of eczema (flexural, or of the cheeks and extensors if under 18 months), history of dry skin in the last year, and visible flexural eczema (or visible eczema of the cheeks and extensors if under 18 months). A personal history of atopy was omitted as a criterion given the young age of the infants, who were not old enough to have developed clearly defined asthma or hay fever.

The trial was approved by the Southampton and South West Hampshire Research Ethics Committee. MAVIDOS was registered prospectively (ISRCTN 82927713 and EudraCT 2007‐001716‐23). Written informed consent was obtained from all parents.

### Statistical analysis

Participant characteristics are described separately for mothers who received cholecalciferol 1000 IU and those who received placebo using frequency and percentage distribution for categorical variables, mean (SD) for normally distributed continuous variables and median (interquartile range) for non‐normally distributed continuous variables.

We used logistic regression to examine associations between being randomized to the active group and developing eczema at ages 12, 24 and 48 months, expressing results as odds ratios (ORs) and 95% confidence intervals (CIs). Models were adjusted for duration of breastfeeding, as descriptive analyses showed differences in breastfeeding duration between the groups. The overwhelming majority of both mothers (Table 1) and their offspring were of white ethnicity, so this was not considered further in stratification and sensitivity analyses. Sensitivity analyses were undertaken to examine whether the effect of the intervention differed in mothers breastfeeding for < 1 month and ≥ 1 month because of its reported association with eczema and the influence of gestational supplementation on breast milk vitamin D content, and to take account of season of birth.

**Table 1:** Characteristics of mothers and offspring with eczema (*n* = 703)

	Placebo	Cholecalciferol (1000 IU daily)
Number	351	352
Maternal characteristics		
Age (years), mean (SD)	31·1 (5·0)	31·0 (4·9)
Ethnicity white	95·8%	95·2%
Parity, nulliparous	42·9%	44·1%
Smoking in early pregnancy	7·0%	5·3%
Educational attainment ≥ A level	76·9%	78·9%
Body mass index (kg m^−2^), median (IQR)	25·4 (22·8–29·4)	24·6 (22·2–28·2)
Sum of all skinfold thicknesses (mm), mean (SD)	81·4 (27·3)	77·8 (28·4)
Early‐pregnancy 25(OH)D (nmol L^−1^), mean (SD)	44·7 (16·2)	46·0 (16·4)
Late‐pregnancy 25(OH)D (nmol L^−1^), mean (SD)	42·4 (20·8)	67·4 (19·9)
Change in 25(OH)D from early to late pregnancy (nmol L^−1^), mean (SD)	−2·0 (20·7)	21·4 (21·9)
Offspring characteristics		
Male	50·1%	55·7%
Birth weight (g), mean (SD)	3543 (495)	3509 (536)
Age last breastfed (months), median (IQR)	4·0 (0–9·0)	5·0 (1·0–10·0)

25(OH)D, 25‐hydroxyvitamin D; IQR, interquartile range.

We additionally used logistic regression to examine the associations between the offspring’s risk of atopic eczema at ages 12, 24 and 48 months and both maternal late‐pregnancy serum 25(OH)D concentration and single‐nucleotide polymorphisms in or near key vitamin D metabolism genes, specifically rs12785878 (*DHCR7*, encoding 7‐dehydrocholesterol reductase in the epidermal vitamin D biosynthesis pathway), rs10741657 (*CYP2R1*, encoding 25‐hydroxylase) and rs6013897 (*CYP24A1*, encoding 24‐hydroxylase).^10^ Analyses were adjusted for characteristics unbalanced between the intervention and placebo groups, and were performed using Stata version 15·1 (StataCorp, College Station, TX, USA).

## Results

### Cohort characteristics

The mother and infant characteristics of the 703 offspring (352 intervention group and 351 placebo) with data on atopic eczema at any of ages 12 (*n* = 635), 24 (*n* = 610) or 48 (*n* = 449) months were similar in the intervention and placebo groups, with the exception of longer breastfeeding duration in the intervention group (Table 1). Mother and infant characteristics apart from breastfeeding duration were also similar for the subgroups followed up at each of the three postnatal ages (Table S1; see Supporting Information). Also, the 703 mothers and offspring included had characteristics similar to those of the overall group recruited to MAVIDOS (Table S2; see Supporting Information). Baseline maternal serum 25(OH)D levels at recruitment in early pregnancy were similar in the intervention and placebo groups. In late pregnancy, maternal serum 25(OH)D levels were higher in the intervention group than in the placebo group (Table 1).

### Association between maternal cholecalciferol supplementation and offspring atopic eczema

**Figure 1:**
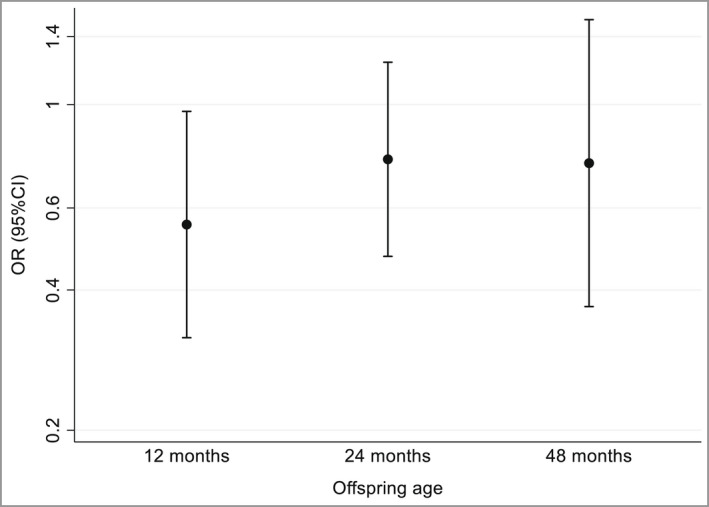
Offspring odds ratios (ORs) with 95% confidence intervals (CIs) of atopic eczema at ages 12, 24 and 48 months in the intervention group whose mothers received cholecalciferol 1000 IU daily during pregnancy vs. the placebo group.

The prevalences of atopic eczema in the intervention group at ages 12, 24 and 48 months were 7·2%, 11·4% and 6·7%, respectively, compared with 12·0%, 14·6% and 8·4% in the placebo group. Table 2 shows the ORs of atopic eczema at ages 12, 24 and 48 months in offspring whose mothers received cholecalciferol 1000 IU vs. placebo. In an unadjusted analysis, offspring of mothers who received cholecalciferol 1000 IU had a lower OR of atopic eczema at age 12 months (OR 0·57, 95% CI 0·33–0·98, *P* = 0·04) than offspring of mothers who received placebo; this changed little after adjusting for breastfeeding duration as a covariate. The ORs of atopic eczema in the intervention group compared with the control group at ages 24 and 48 months were 0·75 (95% CI 0·47–1·21, *P* = 0·24) and 0·79 (95% CI 0·39–1·59, *P* = 0·50), respectively, and changed little in models adjusted for breastfeeding (Table 2) or season at birth (data not shown). The adjusted estimates are presented graphically in Figure 1.

**Table 2:** Offspring atopic eczema in the intervention group compared with the control group

Outcome: atopic eczema	Placebo, *n*/*N* (%)	Cholecalciferol, *n*/*N* (%)	OR (95% CI)	*P*‐value
12 months	38/316 (12·0)	23/319 (7·2)		
Unadjusted			0·57 (0·33–0·98)	0·04
Adjusted^a^			0·55 (0·32–0·97)	0·04
24 months	44/302 (14·6)	35/308 (11·4)		
Unadjusted			0·75 (0·47–1·21)	0·24
Adjusted^a^			0·76 (0·47–1·23)	0·27
48 months	19/226 (8·4)	15/223 (6·7)		
Unadjusted			0·79 (0·39–1·59)	0·50
Adjusted^a^			0·75 (0·37–1·52)	0·42

CI, confidence interval; OR, odds ratio. ^a^Estimates adjusted for breastfeeding duration.

Sensitivity analysis stratified by breastfeeding duration demonstrated a reduced risk of atopic eczema at age 12 months in the intervention group in infants who were breastfed for > 1 month (OR 0·48, 95% CI 0·24–0·93, *P* = 0·03), but not in those who were breastfed for < 1 month (OR 0·80, 95% CI 0·29–2·17, *P* = 0·66) (Table 3). However, interaction terms between the intervention and breastfeeding duration were not statistically significant at any of the three follow‐up ages (*P* = 0·40, 0·35 and 0·15 at ages 12, 24 and 48 months, respectively).

**Table 3:** Offspring atopic eczema in the intervention group, stratified by breastfeeding duration

Outcome: atopic eczema	12 months	24 months	48 months
Breastfed up to 1 month			
Number	248	227	164
Placebo, *n*/*N* (%)	10/133 (7·5)	15/123 (12·2)	4/93 (4)
Cholecalciferol, *n*/*N* (%)	7/115 (6·1)	13/104 (12·5)	5/71 (7)
OR (95% CI)	0·80 (0·29–2·17)	1·03 (0·47–2·27)	1·69 (0·44–6·52)
*P*‐value	0·66	0·95	0·45
Breastfed more than 1 month			
Number	373	369	274
Placebo, *n*/*N* (%)	26/176 (14·8)	28/171 (16·4)	15/125 (12·0)
Cholecalciferol, *n*/*N* (%)	15/197 (7·6)	22/198 (11·1)	10/149 (6·7)
OR (95% CI)	0·48 (0·24–0·93)	0·64 (0·35–1·16)	0·53 (0·23–1·22)
*P*‐value	0·03	0·14	0·14

CI, confidence interval; OR, odds ratio.

Sensitivity analyses showed no association between maternal late‐pregnancy serum 25(OH)D and offspring atopic eczema at any age (Table S3; see Supporting Information). The single‐nucleotide polymorphisms rs12785878 (*DHCR7*), rs10741657 (*CYP2R1*) and rs6013897 (*CYP24A1*) were examined, located in genes involved in vitamin D metabolism.^13^, ^17^ This demonstrated no associations with offspring atopic eczema, although there are indications of higher ORs for atopic eczema with rs10741657 (*CYP2R1*) at ages 12 and 24 months: OR 1·42 (95% CI 0·95–2·12, *P* = 0·09) and OR 1·37 (95% CI 0·95–1·96, *P* = 0·09), respectively (Table 4).

**Table 4:** Single‐nucleotide polymorphism (SNP) associations with offspring atopic eczema at ages 12, 24 and 48 months

Single‐nucleotide polymorphism	Reference allele	Univariate			Adjusted for breastfeeding		
		*n*	OR (95% CI)	*P*‐value	*n*	OR (95% CI)	*P*‐value
Atopic eczema at 12 months							
rs12785878 (*DHCR7*)	G	620	0·86 (0·56–1·32)	0·49	606	0·89 (0·57–1·38)	0·60
rs10741657 (*CYP2R1*)	A	612	1·42 (0·95–2·12)	0·09	598	1·44 (0·95–2·18)	0·08
rs6013897 (*CYP24A1*)	A	614	0·78 (0·50–1·22)	0·28	600	0·75 (0·48–1·19)	0·23
Atopic eczema at 24 months							
rs12785878 (*DHCR7*)	G	593	1·00 (0·67–1·51)	0·98	579	0·97 (0·65–1·47)	0·90
rs10741657 (*CYP2R1*)	A	586	1·37 (0·95–1·96)	0·09	572	1·42 (0·98–2·04)	0·06
rs6013897 (*CYP24A1*)	A	589	0·96 (0·63–1·46)	0·84	575	0·97 (0·63–1·49)	0·89
Atopic eczema at 48 months							
rs12785878 (*DHCR7*)	G	437	1·25 (0·66–2·37)	0·50	427	1·27 (0·67–2·42)	0·46
rs10741657 (*CYP2R1*)	A	429	1·12 (0·67–1·88)	0·66	419	1·14 (0·68–1·92)	0·61
rs6013897 (*CYP24A1*)	A	433	0·82 (0·44–1·50)	0·51	423	0·79 (0·43–1·46)	0·45

CI, confidence interval; OR, odds ratio (per risk allele).

## Discussion

Maternal supplementation with cholecalciferol 1000 IU daily from 14 weeks’ gestation until delivery was associated with a reduced OR of infant atopic eczema at age 12 months. However, the effects of supplementation were nonsignificant at ages 24 and 48 months. Interaction terms between supplementation during pregnancy and breastfeeding duration were not statistically significant, but sensitivity analysis showed that the protective effect of maternal cholecalciferol supplementation on infantile eczema was significant only in offspring breastfed for > 1 month.

Current evidence relating to maternal vitamin D status and its effect on offspring atopic eczema is inconsistent, and evidence from supplementation trials is sparse. A U‐shaped association between maternal vitamin D supply and status and offspring atopic eczema is plausible (see below), whereby both low and high intakes, and 25(OH)D insufficiency and high 25(OH)D concentrations might be associated with increased risk of atopic eczema.^9^, ^18^ We would note that mothers with serum 25(OH)D levels > 100 mmol L^−1^ at baseline were excluded from MAVIDOS, but we found no evidence for an increased risk of atopic eczema with 1000 IU daily cholecalciferol supplementation. A reduced risk of wheeze and eczema has been reported in children of mothers who consumed ≥ 174 IU daily dietary vitamin D during pregnancy,^19^ and infants with cord blood 25(OH)D levels ≥ 75 nmol L^−1^ were found to have a lower risk of eczema in infancy than those with cord blood levels < 50 nmol L^−1^.^20^ There are also reports of no association between maternal or cord serum 25(OH)D concentrations and atopic eczema.^21^ Other studies have reported no clear associations between maternal vitamin D status in late pregnancy and asthma, wheeze or skin sensitization at age 1, 3 or 6 years.^22^

In the VDAART randomized controlled trial in women at high risk of having children with asthma, the prevalence of offspring asthma at age 6 years was similar in those whose mothers received antenatal supplementation with 4400 vs. 400 IU daily vitamin D, as were the secondary outcomes of eczema and total IgE levels.^10^, ^23^, ^24^ However, a between‐group reduction in asthma and recurrent wheeze was suggested at early timepoints through the age of 3 years.^10^, ^23^, ^24^ Our study examined the effect of gestational supplementation on offspring eczema at ages from 12 to 48 months, also finding an effect at an early age, 12 months. We found no effect at 24 and 48 months, suggesting that other, postnatal influences might become important at older ages in affecting the risk of atopic eczema beyond infancy. Conceivably, supplementation during the postnatal period may be needed for a sustained effect. There is evidence supportive of postnatal vitamin D supplementation, with a meta‐analysis of 11 intervention studies in children with atopic eczema reporting a reduction in eczema severity.^25^ The meta‐analysis included no trials in infants, but the included study in children with eczema found a 23‐point improvement in Scoring Atopic Dermatitis following 3 months of vitamin D oral supplementation of 1000 IU daily.^26^

Vitamin D has immunomodulatory effects on innate and adaptive responses.^27^ The vast majority of cells of the adaptive immune system express the vitamin D receptor and CYP27B1, enabling the production of the active metabolite 1,25‐dihydroxyvitamin D3, thought to act predominantly in an autocrine and paracrine fashion.^28^ Evidence from *in vitro* and *in vivo* studies^29^ has demonstrated that vitamin D supplementation inhibits expression of T helper (Th)2 response cytokines, the predominant immune response seen acutely in atopic eczema and other allergic disease.^30^, ^31^ Vitamin D deficiency *in utero* and in early life has been linked with increased Th2 lymphocytes and reduced T regulatory cells and interleukin (IL)‐10, leading to macrophages and dendritic cells producing proinflammatory cytokines.^32^ However, contrary to this, there is evidence that 1,25‐dihydroxyvitamin D3 promotes Th2 responses, with inhibition of interferon‐γ and promotion of IL‐4, IL‐5 and IL‐10 production.^33^ Skin barrier function is important in the pathogenesis of atopic eczema. Vitamin D and its metabolites can impact this through involvement in the synthesis of proteins such as filaggrin, and through stratum corneum formation, keratinocyte formation and differentiation, and production and regulation of skin antimicrobial peptides.^34^, ^35^

Our data suggest that the effect of vitamin D supplementation on offspring eczema risk may be seen soon after pregnancy, but it weakens as children grow older, where other risk factors can be influential. We speculate that during infancy there may be a role of breast milk vitamin D content. Evidence from MAVIDOS has demonstrated an increase in maternal serum levels with 1000 IU daily cholecalciferol supplementation,^15^ but in line with the Southampton Women’s Survey observational study,^22^ our data showed no association between maternal serum 25(OH)D in late pregnancy and offspring atopic eczema. Previous studies have shown that gestational vitamin D supplementation increases breast milk vitamin D content,^11^, ^12^ and in MAVIDOS the vitamin D content of breast milk is likely to have been higher in the supplemented group, influenced by mobilization from maternal fat and muscle tissue. This may explain our finding of a protective effect only in children who were breastfed for > 1 month. Heterogeneity in the aetiology and pathogenesis of atopic eczema in early childhood is increasingly recognized,^36^ and an alternative possibility is that vitamin D supplementation may only have an effect on particular atopic eczema phenotypes.

In MAVIDOS, examination of genetic variants in genes related to the vitamin D pathway has shown that rs12785878 (*DHCR7*) was associated with baseline 25(OH)D, probably influencing cutaneous synthesis. Achieved 25(OH)D status following supplementation was associated with rs10741657 [*CYP2R1*, which determines the efficiency of vitamin D to 25(OH)D conversion], whereas rs12785878 and rs6013897 (*CYP24A1*) were not.^13^ We found trends for higher ORs of atopic eczema with rs10741657 (*CYP2R1*) at ages 12 and 24 months but no associations for the other single‐nucleotide polymorphisms examined. In a case–control study of Chinese children, rs4674343 on *CYP27A1* (27‐hydroxylase, an enzyme converting the pre‐vitamin D3 metabolite lumisterol into further downstream metabolites with biological activity in skin cells)^37^ was reported to be protective against atopic eczema, and *CYP2R1* and *VDR* haplotypes also influenced atopic eczema risk and eosinophil count.^38^

A strength of this study is analysis of data from a placebo‐controlled, double‐blind, randomized trial. Atopic eczema was not the primary outcome in MAVIDOS, but the data collected enabled ascertainment of offspring atopic eczema using the UK Working Party’s well‐recognized criteria for the diagnosis of atopic eczema. Furthermore, these criteria were determined by trained research nurses who examined the offspring. While some participants were taking vitamin D in addition to the intervention or placebo provided, supplement use at interview did not differ between the intervention and placebo groups. Additionally, maternal diagnosis of eczema did not differ between the two groups. The number of offspring assessed for eczema was lower at age 48 months than at age 12 months, lowering the statistical power to identify a significant effect of prenatal intervention on early childhood eczema. Cord blood and offspring 25(OH)D levels were not measured, precluding examination of these in relation to atopic eczema. Possible effects of ultraviolet B and any interaction with supplementation could not be investigated because no ultraviolet B exposure data were available.

In conclusion, in a randomized controlled trial, maternal supplementation with cholecalciferol 1000 IU daily from 14 weeks’ gestation to delivery led to a reduced incidence of atopic eczema in the first year of life. Many international and national guidelines recommend cholecalciferol 400–600 IU daily (10–15 μg) throughout pregnancy, with the strongest evidence for the prevention of neonatal hypocalcaemia and emerging evidence for effects on other health outcomes affecting the skeletal, respiratory and immune systems.^39^ The current findings inform understanding of the early‐life influences on infantile eczema and support recommendations for routine vitamin D supplementation during pregnancy.

## Funding sources

This work was supported by grants from Arthritis Research UK (17702), Medical Research Council (4050502589), Bupa Foundation, National Institute for Health Research (NIHR) Southampton Biomedical Research Centre, University of Southampton and University Hospital Southampton NHS Foundation Trust, and NIHR Musculoskeletal Biomedical Research Unit, University of Oxford. Inez Schoenmakers and Ann Prentice (MAVIDOS Trial Group) were funded by the MRC (programme code U105960371). K.M.G. is supported by the UK Medical Research Council (MC_UU_12011/4), the National Institute for Health Research (Senior Investigator; NF‐SI‐0515‐10042), NIHR Southampton 1000DaysPlus Global Nutrition Research Group (17/63/154), NIHR Southampton Biomedical Research Centre (IS‐BRC‐1215‐20004), the European Union (Erasmus+ Programme ImpENSA 598488‐EPP‐1‐2018‐1‐DE‐EPPKA2‐CBHE‐JP) and the British Heart Foundation (RG/15/17/3174). The work leading to these results was supported by the European Union’s Seventh Framework Programme (FP7/2007‐2013), projects EarlyNutrition and ODIN under grant agreements numbers 289346 and 613977, and by the BBSRC (HDHL‐Biomarkers, BB/P028179/1), as part of the ALPHABET project, supported by an award made through the ERA‐Net on Biomarkers for Nutrition and Health (ERA HDHL), Horizon 2020 grant agreement number 696295. We are extremely grateful to Merck GmbH for the kind provision of the Vigantoletten supplement. Merck GmbH had no role in the trial execution, data collection, analysis or manuscript preparation.

## Conflicts of interest

K.M.G. has received reimbursement for speaking at conferences sponsored by companies selling nutritional products unrelated to the vitamin D supplement trialled in this study; and is part of an academic consortium that has received research funding from Abbott Nutrition, Nestec and Danone. C.C. reports personal fees from Alliance for Better Bone Health, Amgen, Eli Lilly, GSK, Medtronic, Merck, Novartis, Pfizer, Roche, Servier and Takeda, outside the submitted work. N.C.H. reports personal fees, consultancy fees, lecture fees and honoraria from Alliance for Better Bone Health, Amgen, MSD, Eli Lilly, Servier, Shire, Radius Health, UCB, Consilient Healthcare and Internis Pharma, outside the submitted work. The other authors declare they have no conflicts of interest.

## Author contributions

**Sarah El‐Heis:** Conceptualization (lead); formal analysis (equal); methodology (lead); project administration (lead); writing – original draft (lead); writing – review and editing (lead). **Stefania D'Angelo:** Formal analysis (equal); methodology (equal); writing – original draft (equal); writing – review and editing (equal). **Elizabeth M Curtis:** Data curation (supporting); writing – review and editing (supporting). **Eugene Healy:** Supervision (supporting); writing – review and editing (supporting). **Rebecca J Moon:** Data curation (supporting); writing – review and editing (supporting). **Sarah R Crozier:** Formal analysis (supporting); methodology (supporting); writing – review and editing (supporting). **Hazel Inskip:** Formal analysis (supporting); methodology (supporting); supervision (supporting); writing – review and editing (supporting). **Cyrus Cooper:** Funding acquisition (equal); supervision (supporting); writing – review and editing (supporting). **Nicholas C Harvey:** Funding acquisition (equal); supervision (supporting); writing – review and editing (supporting). **Keith M Godfrey:** Conceptualization (supporting); funding acquisition (equal); methodology (supporting); project administration (supporting); supervision (equal); writing – review and editing (supporting).

## Ethics statement

The trial was approved by the Southampton and South West Hampshire Research Ethics Committee. MAVIDOS was registered prospectively (ISRCTN 82927713; EudraCT 2007‐001716‐23). Written informed consent was obtained from all parents.

## Supporting information


**Figure S1** Consort diagram.
**Table S1** Characteristics of mothers and offspring.
**Table S2** Baseline characteristics of the 703 mothers and offspring included in the study compared with the full MAVIDOS sample.
**Table S3** Association between late‐pregnancy maternal serum 25‐hydroxyvitamin D (nmol L^−1^) and offspring eczema.

## Data Availability

Requests for data sharing should be directed to Professor Cyrus Cooper, Director, MRC Lifecourse Epidemiology Centre.
