# Supplementary material for: Maternal antenatal vitamin D supplementation and offspring risk of atopic eczema in the first 4 years of life: evidence from a randomized controlled trial
Source: Br J Dermatol. 2022 Aug 3;187(5):659–66. doi: 10.1111/bjd.21721 (PMC9804289; doi:10.1111/bjd.21721)
Supplement: Supplementary file 1 — Figure S1 Consort diagram. Table S1 Characteristics of mothers and offspring. Table S2 Baseline characteristics of the 703 mothers and offspring included in the study compared with the full MAVIDOS sample. Table S3 Association between late‐pregnancy maternal serum 25‐hydroxyvitamin D (nmol L−1) and offspring eczema. [file BJD-187-659-s001.docx]

Figure S1. Consort diagram

ECZEMA DATA AT 12/24 OR 48 MONTHS

N=351

Missing data on eczema at 12 months N=170

Missing data on eczema at 24 months N=184

Missing data on eczema at 48 months N=260

BIRTHS

N=486

RANDOMISED TO CONTROL GROUP

N=569

Withdrawn N=62

Miscarriage/clinical complication N=15

Vit D supplementation >400 iu/day N=6

ECZEMA DATA AT 12 MONTHS

N=319

ECZEMA DATA AT 24 MONTHS

N=308

ECZEMA DATA AT 48 MONTHS

N=223

Missing data on eczema at 12 months N=160

Missing data on eczema at 24 months N=171

Missing data on eczema at 48 months N=256

Withdrawn N=72

Miscarriage/clinical complication N=4

Vit D supplementation >400 iu/day N=10

ECZEMA DATA AT 12/24 OR 48 MONTHS

N=352

BIRTHS

N=479

RANDOMISED TO INTERVENTION GROUP

N=565

RANDOMISED

N=1134

SCREENED N= 1449

High Vit D N=59

Low Vit D N=89

Withdrawn N=167

ECZEMA DATA AT 48 MONTHS

N=226

ECZEMA DATA AT 24 MONTHS

N=302

ECZEMA DATA AT 12 MONTHS

N=316

Table S1. Characteristics of mothers and offspring

|  | 12 months | | 24 months | | 48 months | |
| --- | --- | --- | --- | --- | --- | --- |
|  | **Placebo** | **Cholecalciferol (1000 IU/day)** | **Placebo** | **Cholecalciferol (1000 IU/day)** | **Placebo** | **Cholecalciferol (1000 IU/day)** |
| N | 316 | 319 | 302 | 308 | 226 | 223 |
| *Maternal characteristics* |  |  |  |  |  |  |
| Age (years), mean (SD) | 31.2 (5.0) | 31.1 (4.7) | 31.1 (5.0) | 31.1 (5.0) | 31.4 (4.9) | 31.6 (4.7) |
| Ethnicity, % Caucasian | 95.4% | 96.1% | 96.5% | 95.2% | 98.1% | 94.7% |
| Parity, % nulliparous | 42.8% | 44.8% | 44.3% | 43.6% | 44.5% | 43.3% |
| Smoking in early pregnancy, % | 5.7% | 5.6% | 7.4% | 4.7% | 6.2% | 4.8% |
| Educational attainment ≥ A level, % | 77.0% | 80.0% | 77.3% | 80.1% | 79.8% | 84.8% |
| BMI (kg/m^2^), median (IQR) | 25.5 (23.0,29.6) | 24.5 (22.2,27.9) | 25.1 (22.8,29.2) | 24.9 (22.2,28.3) | 25.1 (22.8,29.0) | 24.8 (22.4,28.6) |
| Sum of all skinfold thicknesses (mm), mean±SD | 82.1 (27.4) | 77.9 (28.2) | 80.7 (27.1) | 78.9 (28.8) | 80.6 (27.8) | 78.1 (29.3) |
| Early pregnancy 25(OH)D (nmol/l), mean (SD) | 44.5 (16.1) | 46.3 (16.5) | 44.9 (16.0) | 45.2 (16.2) | 45.6 (16.1) | 44.6 (16.3) |
| Late pregnancy 25(OH)D (nmol/l), mean (SD) | 42.9 (20.7) | 67.5 (19.7) | 41.9 (20.6) | 66.4 (19.7) | 42.3 (21.6) | 67.9 (19.4) |
| Change in 25(OH)D from early to late pregnancy (nmol/l), mean (SD) | -1.4 (20.5) | 21.2 (22.4) | -2.9 (20.0) | 21.2 (22.1) | -3.3 (20.4) | 23.1 (20.7) |
|  |  |  |  |  |  |  |
| *Children's characteristics* |  |  |  |  |  |  |
| Male, (%) | 49.1% | 55.8% | 50.7% | 57.1% | 49.1% | 56.1% |
| Birth weight (g), mean(SD) | 3537.6 (496.2) | 3515.3 (529.1) | 3528.7 (493.2) | 3531.3 (516.2) | 3552.4 (479.1) | 3556.8 (537.9) |
| Age (years), mean(SD) | 1.1 (0.1) | 1.1 (0.1) | 2.1 (0.1) | 2.0 (0.1) | 4.1 (0.1) | 4.1 (0.1) |
| Age last breastfed (months), median (IQR) | 3.5 (0,9.0) | 5.0 (1.0,10.0) | 4.0 (0,9.0) | 5.0 (1.0,11.0) | 3.0 (0,8.0) | 6.0 (1.0,11.0) |
| Atopic eczema, % | 12.0% | 7.2% | 14.6% | 11.4% | 8.4% | 6.7% |

Table S2. Baseline characteristics of the 703 mother/offspring included in the study compared with the full MAVIDOS sample

|  | **Sample included in the analysis** | **Full MAVIDOS sample** |
| --- | --- | --- |
| N | 703 | 1134 |
| *Maternal characteristics* |  |  |
| Age (years), mean (SD) | 31.0 (5.0) | 30.5 (5.2) |
| Ethnicity, % Caucasian | 95.5% | 94.1% |
| Parity, % nulliparous | 43.5% | 43.8% |
| Smoking in early pregnancy, % | 6.1% | 8.2% |
| Educational attainment ≥ A level, % | 77.9% | 76.6% |
| Walking speed at least fairly brisk, % | 39.7% | 39.6% |
| Strenuous exercise ≥ once week, % | 15.5% | 14.9% |
| Height (cm), mean (SD) | 165.7 (6.3) | 165.7 (6.5) |
| Weight (kg), median (IQR) | 71.8 (13.9) | 72.5 (14.7) |
| BMI (kg/m^2^), median (IQR) | 25.0 (22.5,28.9) | 25.1 (22.6,29.5) |
| Sum of all skinfold thicknesses (mm), mean (SD) | 79.5 (27.9) | 81.9 (27.9) |
| Early pregnancy 25(OH)D (nmol/l), mean (SD) | 45.3 (16.3) | 46.3 (17.4) |
| Late pregnancy 25(OH)D (nmol/l), mean (SD) | 54.8 (23.9) | 55.3 (25.3) |
| Change in 25(OH)D from early to late pregnancy (nmol/l), mean (SD) | 9.6 (24.3) | 9.0 (26.4) |
|  |  |  |
| *Children's characteristics* |  |  |
| Male, (%) | 52.9% | 52.8% |
| Birth weight (g), mean(SD) | 3526 (516) | 3499 (530) |
| Age last breastfed (months), median (IQR) | 4 (1,10) | 4 (4,10) |

Table S3. Association between late pregnancy maternal serum 25(OH)D (nmol/l) and offspring eczema

|  | **Overall** | | | **Placebo** | | | **1000 IU/day cholecalciferol** | | |
| --- | --- | --- | --- | --- | --- | --- | --- | --- | --- |
| Outcome: Atopic eczema | N | OR (95%CI) | p | N | OR (95%CI) | p | N | OR (95%CI) | p |
| 12 months | 561 | 1.00 (0.99,1.01) | 0.82 | 281 | 1.00 (0.99,1.02) | 0.65 | 280 | 1.01 (0.99,1.03) | 0.51 |
| 24 months | 542 | 1.00 (0.99,1.01) | 0.92 | 270 | 1.01 (0.99,1.02) | 0.36 | 272 | 1.01 (0.99,1.03) | 0.47 |
| 48 months | 430 | 1.00 (0.99,1.02) | 0.93 | 217 | 1.01 (0.99,1.03) | 0.37 | 213 | 1.00 (0.97,1.03) | 0.90 |
